# Impact of Insulin Resistance on Cardiometabolic Risk Factors and an Anthropometry-Based Predictive Nomogram for Insulin Resistance Among Adolescents in China

**DOI:** 10.3389/fendo.2022.852395

**Published:** 2022-03-28

**Authors:** Runyu Du, Ling Li, Ping Li, Yanjun Wang

**Affiliations:** Department of Endocrinology, Shengjing Hospital of China Medical University, Shenyang, China

**Keywords:** adolescent, anthropometry, cardiometabolic risk factors, China, homeostasis model assessment, insulin resistance, nomogram

## Abstract

**Objective:**

We aimed to investigate the impact of insulin resistance (IR), as determined by the homeostasis model assessment of insulin resistance (HOMA-IR), on cardiometabolic risk factors (CMRFs), and develop an anthropometry-based predictive nomogram for IR among adolescents in China.

**Design:**

Data were acquired from a cross-sectional study with a stratified cluster sampling method, conducted among adolescents in Northeast China.

**Participants:**

A total of 882 adolescents (aged 12–16 years, 468 boys) were included.

**Measurements:**

All participants underwent anthropometric and biochemical examinations. The thresholds of IR included the 90th percentile of the HOMA-IR for adolescents with a normal body mass index (BMI) and fasting plasma glucose (FPG) level within each sex group (Cutoff A), and the 75th percentile for all participants of the same sex (Cutoff B).

**Results:**

The HOMA-IR was associated with CMRFs. IR, as defined by both cutoffs A and B, was significantly associated with most CMRFs, except decreased HDL-C levels. Excellent concordance (κ = 0.825) was found between these two criteria in diagnosing IR. However, IR using cutoff A, was more closely associated with cardiometabolic risk. The incidence of IR, as defined by cutoff A, was 18.93% and increased from 10.99% to 43.87% based on the different BMI categories. Further, an anthropometry-based predictive model for IR, incorporating sex, age, waist-to-hip ratio, weight and BMI, was developed and presented as a nomogram.

**Conclusions:**

IR among adolescents is strongly related to cardiometabolic risk. We developed an anthropometry-based predictive nomogram for IR among adolescents, which may facilitate health counselling and self-risk assessments.

## Introduction

Insulin resistance (IR) is often associated with the cluster of metabolically related cardiovascular risk factors [cardiometabolic risk factors (CMRFs)] that lead to morbidity and mortality worldwide (1–3). Owing to the global epidemic of obesity among children and adolescents, IR is also present in early life and contributes to metabolic disorders in childhood that tend to persist into adulthood (1). It is difficult to directly measure the incidence of IR due to the practical, ethical and economic issues involved with using the hyperinsulinemic-euglycemic clamp, which is considered the gold standard for measuring insulin sensitivity (4). The homeostasis model assessment of IR (HOMA-IR) has been proposed as a robust surrogate method for classifying IR in epidemiological studies (5, 6), and has been found to be more reliable than the fasting plasma glucose-to-insulin ratio and quantitative insulin sensitivity check index for quantifying the degree of IR in children with obesity (7). Additionally, puberty has also been associated with marked IR, and is known as “physiological IR” (1, 8). Among adults, the universally accepted HOMA-IR cutoff value for IR is 2.5 (5). However, among adolescents, a specific cutoff criterion for defining IR is still urgently needed to avoid under- or over-diagnosis. Here, using the data from a population-based study among adolescents in China, we aimed to classify IR in adolescents based on specific criteria, prevalence, and the extent of influences on CMRFs.

Owing to the use of anthropometric methods being convenient, noninvasive, and inexpensive, the importance of using these for the clinical diagnosis of obesity-related health risks is emphasized (9, 10). Overweight and obesity are frequently associated with IR (1, 2, 11). Weight, body mass index (BMI), waist circumference (WC), waist-to-hip ratio (WHR) and waist-to-height ratio (WHtR) are commonly used anthropometric indices for assessing global or local obesity in individuals. When identifying adolescents at risk of IR, anthropometric methods may be effective if the diagnostic values are appropriately specified in a statistical model. The nomogram is a simple and personalized visualization tool, that has been extensively used in the diagnosis of diseases. Here, we further aimed to develop an anthropometry-based predictive nomogram for early detection of IR among adolescents in China.

## Materials and Methods

### Study Design and Data Collection

Data were acquired from a cross-sectional study that used stratified cluster sampling to include a total of 933 adolescents from the urban district of Liaoyang, a moderately sized city in northeast China. A total of 936 participants without known metabolic syndrome (MetS) were recruited, and 3 of which with HbA1c of ≥ 6.5% or FPG of ≥ 7.0 mmol/L were excluded because of the possibility of a diagnosis of diabetes. Ethical permission from the hospital’s Ethics Committee and written informed consent from the students and their parents were obtained prior to commencing the original study.

The following data were analyzed for the current study: age, sex, weight, height, WC, hip circumference (HC), systolic blood pressure (SBP), diastolic blood pressure (DBP), hemoglobin A1c (HbA1c), fasting plasma glucose (FPG), fasting plasma insulin (FINS), serum uric acid (SUA), total cholesterol (TC), triglycerides (TG), high-density lipoprotein cholesterol (HDL-C), low-density lipoprotein cholesterol (LDL-C), and a family history of type 2 diabetes mellitus (T2DM, defined as having a first- or second-degree relative with T2DM). A total of 882 participants (aged 12–16 years, 468 boys), without missing data on the variables described above, were included in the current analysis.

Physical examinations were conducted by trained physicians. Anthropometric indices were measured in a standing position while lightly clothed and without shoes. Weight was measured to the nearest 0.1 kg and height was measured to the nearest 0.1 cm. WC and HC were measured at the narrowest point between the iliac crest and the lowest rib during minimal respiration and at the largest girth, respectively. SBP and DBP were measured in the sitting position after a 10-minute rest, and calculated as the mean value of two repeated measurements. BMI was calculated as weight divided by height squared (kg/m^2^). WHR and WHtR were calculated as the quotient of the circumference of the waist to that of the hips and WC divided by height, respectively.

Blood samples were collected after an overnight fast (≥10 h). FPG, TG, TC, HDL-C and SUA were measured using an Olympus AU system (Olympus 400, Olympus Optical Company, Japan) in the laboratory at Liaoyang Diabetes Hospital. We further estimated LDL-C using Friedwald’s formula (12). HbA1c levels were measured using high performance liquid chromatography with a D-10 Hemoglobin Testing System (Bio-Rad Laboratories, Inc., Hercules, CA) in the central laboratory at Shengjing Hospital of China Medical University. HbA1c was standardized to the Diabetes Complications and Control Trial method. FINS was determined by radioimmunoassay (China Institute of Atomic Energy, Beijing, China). HOMA-IR was calculated as FINS (µU/mL) × FPG (mmol/L)/22.5 (5).

### Definitions

Obesity was defined as a BMI ≥95th percentile, and overweight was defined as a BMI ≥85th percentile and <95th percentile for children and adolescents of the same age and sex in China (13). Although IR is affected by pubertal status (8), Tanner stage data were not available for use in our study. Boys aged 12-18 years and girls aged 11-18 years are usually regarded as adolescents in China, because pubertal spurt is between ages 12 and 14 in boys and 11 and 13 in girls (14–18); thus, the participants (aged 12–16 years) in our study were all considered to be adolescents. Currently, there is no universally accepted method for defining the optimal threshold for IR among adolescents. Therefore, we adopted the 90th percentile of the HOMA-IR for adolescents with a normal BMI and FPG level within each sex group (Cutoff A) (7, 19), and the 75th percentile for all participants of the same sex (Cutoff B), as the thresholds of IR (20, 21). IR was identified when the HOMA-IR was above the following cutoff points: A: 5.64 (boy), 5.73 (girl) and B: 5.24 (boy), 5.20 (girl). According to the unified definition of pediatric metabolic syndrome (MetS) by the International Diabetes Federation (IDF) (22), MetS was identified in adolescents with abdominal obesity (defined as a WC ≥90th percentile in children and adolescents of the same age and sex in China) (23) and at least two of the following four criteria: TG levels of ≥1.70 mmol/L; HDL-C levels of <1.03 mmol/L for individuals aged 10–15 years and boys aged ≥16 years, or < 1.29 mmol/L for girls aged ≥16 years; an SBP of ≥130 mmHg and/or a DBP of ≥85 mmHg; and a FPG level of ≥5.6 mmol/L or T2DM.

Abdominal obesity, hypertension, increased FPG levels, dyslipidemia and hyperuricemia were assessed as CMRFs in this study. We used the definition of MetS (IDF) as a reference, which includes abdominal obesity, hypertension, increased FPG levels, increased TG levels and decreased HDL-C levels. Since neither the IDF nor the recommendations in China included threshold values for TC and LDL-C in children; we adopted the threshold values of a TC level of ≥5.2 mmol/L and a LDL-C level of ≥3.4 mmol/L as proposed by the American Academy of Pediatrics (24). Dyslipidemia was defined as the presence of at least one of the following: hypercholesterolemia, hypertriglyceridemia, hypo-HDL-C, and hyper-LDL-C. Due to the lack of universally accepted clinical diagnostic criteria for hyperuricemia in adolescents, we defined hyperuricemia as a SUA level of ≥357 µmol/L, which is consistent with similar previous studies (25). Clustering of CMRFs was defined as the presence of two or more CMRFs (26).

### Statistical Analysis

The Shapiro–Wilk and Levene tests were used to assess the normal distribution and homogeneity of each variable, respectively. Quantitative variables are expressed as the mean ± standard deviation or median (interquartile range) depending on the normality of their distributions, whereas qualitative variables are presented as counts and percentages. Quantitative data were analyzed using the Student’s t-test or Mann–Whitney U-test, and qualitative data were analyzed using the Chi-square test. Significance tests for differences in proportions were conducted using the two-tailed two-sample test approach. A partial correlation analysis was used to determine the relationship between HOMA-IR and CMRFs following adjustments for age and sex. We performed binary logistic regression analysis to determine the influence of IR on CMRFs. The kappa coefficient (κ) was calculated to determine the degree of agreement between the different definitions of IR. The prevalence of IR was calculated while considering BMI stratification. A least absolute shrinkage and selection operator (LASSO) regression analysis was used to select the most predictive anthropometric indices for IR (27, 28). Subsequently, a logistic regression analysis was performed to establish a prediction model by introducing the variables based on the LASSO regression, and the nomogram was constructed to achieve individual predictions. To validate the classification abilities of the nomogram, a bootstrapping calibration approach was used and randomly repeated 1000 times with replacement. Decision curve analysis and clinical impact curves were used to determine clinical usefulness. Data processing and statistical analyses were performed using R software (version 4.0.3; R project for Statistical Computing) and *P*-values <0.05 were considered statistically significant.

## Results

### Patient Characteristics

Among the 882 adolescents (468 boys), the median age was 14 (12–15) years, and 69 had MetS. The demographic data, physical examination results, and biochemical characteristics of the study participants according to gender are presented in **Table 1**. Boys had higher values for weight, BMI, WC, WHR, WHtR, SBP, FPG, SUA and HbA1c. Girls had higher values for TC, DBP and insulin levels, while there were no significant differences in values of HOMA-IR or other indices evaluated. Adolescent males were more likely to have abdominal obesity and hyperglycemia, contributing to a higher incidence of MetS.

**Table 1:** Characteristics of the study participants.

	Total (n = 882)	Male (n = 468)	Female (n = 414)
**Age (years)**	14 (12–15)	14 (12–15)	14 (12.25–15)
**Ethnic (Han)**	786 (89.12%)	420 (89.74%)	366 (88.41%)
**WC (cm)**	74.5 (69.5,83.0)	77.25 (70.50–87.50)	73.50 (68.50–78.50)***
**HC (cm)**	93.5 (88.5,99.5)	93.5 (88.5–100.5)	93.5 (88.5–98.5)
**WHR**	0.81 (0.77,0.85)	0.83 (0.79–0.87)	0.79 (0.75–0.83)***
**WHtR**	0.46 (0.43–0.50)	0.47 (0.43–0.52)	0.46 (0.43–0.49)*
**Weight (kg)**	55.5 (48.5–64.5)	59.5 (50.5–72.5)	51.5 (46.5–59.5)***
**BMI (kg/m^2^)**	20.63 (18.55,23.83)	21.23 (18.89–24.95)	19.99 (18.25–22.91)***
**SBP (mmHg)**	117 (109–126)	121(111–130)	115 (107–122)***
**DBP (mmHg)**	72.87 ± 10.78	71.76 ± 11.03	74.12 ± 10.35**
**Family history of T2DM**	487 (55.22%)	255 (54.49%)	232 (56.04%)
**FPG (mmol/L)**	4.7 (4.4–5.0)	4.8 (4.5–5.1)	4.7 (4.4–5.0)**
**TC (mmol/L)**	4.46 (3.92–5.04)	4.35 (3.84–4.92)	4.60 (4.03–5.13)***
**HDL-C (mmol/L)**	1.06 (0.87–1.27)	1.06 (0.85–1.26)	1.06 (0.90–1.29)
**TG (mmol/L)**	0.94 (0.67–1.28)	0.90 (0.65–1.27)	0.96 (0.70–1.30)
**LDL-C (mmol/L)**	3.30 (3.25–3.48)	3.30 (3.25–3.47)	3.31 (3.25–3.49)
**SUA (μmol/l)**	301.50 (248.25,367.00)	347.50 (297.75–412.25)	260.00 (225.25–300.75)***
**HbA1c (%)**	5.4 (5.3–5.6)	5.5 (5.3–5.6)	5.4 (5.2–5.6)**
**FINS (μU/mL)**	18 (13–24)	17.00 (12.00–24.13)	19.00 (14.05–24.00)*
**HOMA-IR**	3.77 (2.72–5.22)	3.71 (2.84–5.23)	3.86 (2.48–5.18)
**MetS**	69 (7.82%)	53 (11.32%)	16 (3.86%)***
** Abdominal obesity**	184 (20.86%)	111 (23.72%)	71 (17.63%)*
** Hypertension**	228 (25.85%)	148 (31.62%)	80 (19.32%)***
** Increased FPG**	56 (6.35%)	36 (7.69%)	20 (4.83%)
** Increased TG**	86 (9.75%)	54 (11.54%)	32 (7.73%)
** Decreased HDL**	432 (48.98%)	230 (49.14%)	202 (48.79%)

Quantitative data are expressed as the mean ± SD or median (25th–75th percentile), and qualitative data as the number (percent).

***P < 0.001, **P < 0.01; *P < 0.05 compared with males.

BMI, body mass index; DBP, diastolic blood pressure; FINS, fasting plasma insulin; FPG, fasting plasma glucose; HbA1c, hemoglobin A1c; HC, hip circumference; HDL-C, high-density lipoprotein cholesterol; HOMA-IR, homeostasis model assessment of insulin resistance; LDL-C, low-density lipoprotein cholesterol; MetS, metabolic syndrome; SBP, systolic blood pressure; SUA, serum uric acid; T2DM, type 2 diabetes mellitus; TC, total cholesterol; TG, triglycerides; WC, waist circumference; WHR, waist-to-hip ratio; WHtR, waist-to-height ratio.

### Association Between Insulin Resistance and Cardiometabolic Risk Factors

**Figure 1:**
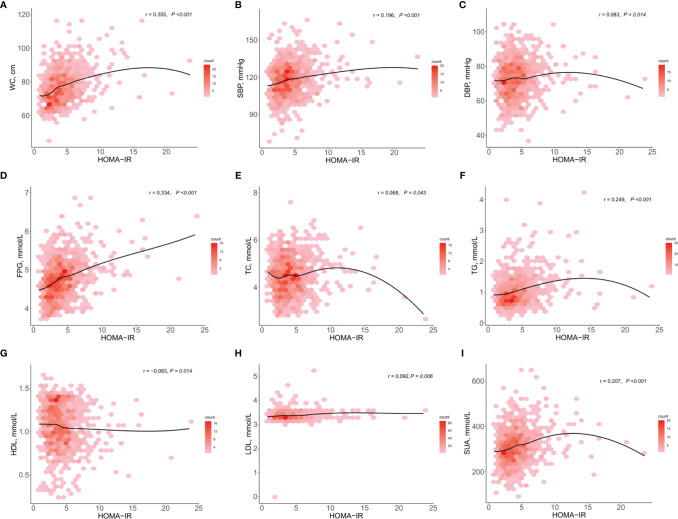
Scatterplots of the homeostasis model assessment of insulin resistance against cardiometabolic risk factors (n = 882). Partial correlation coefficient of the HOMA-IR with WC, SBP, DBP, FPG, TC, TG, HDL, LDL, and SUA **(A–I)**, adjusted for age and sex. Darker red denotes higher density. The black line is a smoothed condition mean, denoting the mean value of the cardiometabolic risk factor at a given HOMA-IR. DBP, diastolic blood pressure; FPG, fasting plasma glucose; HDL-C, high-density lipoprotein cholesterol; LDL-C, low-density lipoprotein cholesterol; SBP, systolic blood pressure; SUA, serum uric acid; TC, total cholesterol; TG, triglycerides; WC, waist circumference.

The HOMA-IR was more significantly correlated with WC, FPG, TG, and SUA (r > 2, P <0.001), compared with other CMRFs evaluated (**Figure 1**). We analyzed the influence of IR, as defined by the different HOMA-IR thresholds (Cutoffs A and B), on CMRFs using a logistic regression analysis (**Table 2**). Whether adjusted for age and sex or not, IR as defined by both cutoffs A and B, was significantly associated with most CMRFs, except decreased HDL-C levels. More specifically, IR, as defined by cutoff B, was not correlated with hypercholesterolemia, and was associated with dyslipidemia only after adjustments for sex and age. Among participants with IR, the cardiometabolic risk increased by 3.132- and 2.662-fold according to cutoffs A and B, respectively. Excellent concordance (κ = 0.825, *P <*0.001) was found between these two criteria in diagnosing IR among adolescents in the Northeast of China (**Table 3**). Owing to the higher odds ratios for CMRFs, IR as defined by cutoff A was chosen for subsequent analyses.

**Table 2:** The influence of insulin resistance on cardiometabolic risk factors according to different definitions of insulin resistance.

Items		Cutoff A	*P*	Cutoff B	*P*
**Abdominal obesity**	Crude OR (95% CI)	3.081 (2.131–4.454)	<0.001	2.972 (2.104–4.199)	<0.001
	Adjusted OR (95% CI)	3.027 (2.090–4.386)	<0.001	2.973 (2.099–4.210)	<0.001
**Hypertension**	Crude OR (95% CI)	1.888 (1.318–2.705)	0.001	1.624 (1.164–2.267)	0.004
	Adjusted OR (95% CI)	1.964 (1.359–2.838)	<0.001	1.724 (1.225–2.425)	0.002
**Increased FPG**	Crude OR (95% CI)	3.885 (2.226–6.780)	<0.001	3.571 (2.063–6.180)	<0.001
	Adjusted OR (95% CI)	3.730 (2.108–6.600)	<0.001	3.468 (1.979–6.077)	<0.001
**Dyslipidemia**	Crude OR (95% CI)	1.538 (1.011–2.339)	0.044	1.380 (0.956–1.994)	0.086
	Adjusted OR (95% CI)	1.697 (1.108–2.600)	0.015	1.495 (1.028-2.174)	0.035
** Increased TC**	Crude OR (95% CI)	1.676 (1.133–2.478)	0.01	1.322 (0.914–1.913)	0.138
	Adjusted OR (95% CI)	1.748 (1.177–2.596)	0.006	1.345 (0.927–1.951)	0.119
** Hypertriglyceridemia**	Crude OR (95% CI)	2.735 (1.697–4.409)	<0.001	2.395 (1.513–3.790)	<0.001
	Adjusted OR (95% CI)	2.624 (1.601–4.300)	<0.001	2.311 (1.439–3.713)	0.001
** Increased LDL-C**	Crude OR (95% CI)	1.536 (1.091–2.163)	0.014	1.534 (1.124-2.095)	0.007
	Adjusted OR (95% CI)	1.478 (1.044–2.092)	0.028	1.467 (1.070-2.013)	0.017
** Decreased HDL-C**	Crude OR (95% CI)	1.067 (0.762–1.495)	0.705	1.031 (0.760–1.398)	0.846
	Adjusted OR (95% CI)	1.245 (0.871–1.780)	0.229	1.186 (0.857–1.640)	0.304
**Hyperuricemia**	Crude OR (95% CI)	2.193 (1.544–3.116)	<0.001	1.694 (1.223–2.346)	0.002
	Adjusted OR (95% CI)	2.465 (1.647–3.691)	<0.001	1.974 (1.361–2.863)	<0.001
**Clustering of cardiometabolic risk factors**	Crude OR (95% CI)	2.861 (2.010–4.073)	<0.001	2.349 (1.719–3.210)	<0.001
	Adjusted OR (95% CI)	3.132 (2.160–4.540)	<0.001	2.662 (1.913–3.705)	<0.001

Crude OR is the OR before adjusting for sex and age; adjusted OR is the OR after adjusting for sex and age. Clustering of cardiometabolic risk factors: defined as ≥2 cardiometabolic risk factors.

CI, confidence interval; FPG, fasting plasma glucose; HDL-C, high-density lipoprotein cholesterol; LDL-C, low-density lipoprotein cholesterol; OR, odds ratio; TC, total cholesterol.

**Table 3:** Concordance between the different homeostasis model assessment of insulin resistance cutoff values used in diagnosing insulin resistance.

	IR diagnosed by cutoff B				κ (95% CI)	*P*
		-	+	Total		
**IR diagnosed by cutoff A**	**-**	662	53	715	0.825 (0.780, 0.870)	<0.001
	**+**	0	167	167		
	**Total**	662	220	882		

+ with IR; − without IR.

IR, insulin resistance; CI, confidence interval.

### The Incidence of Insulin Resistance Based on Body Mass Index Categories

**Figure 2:**
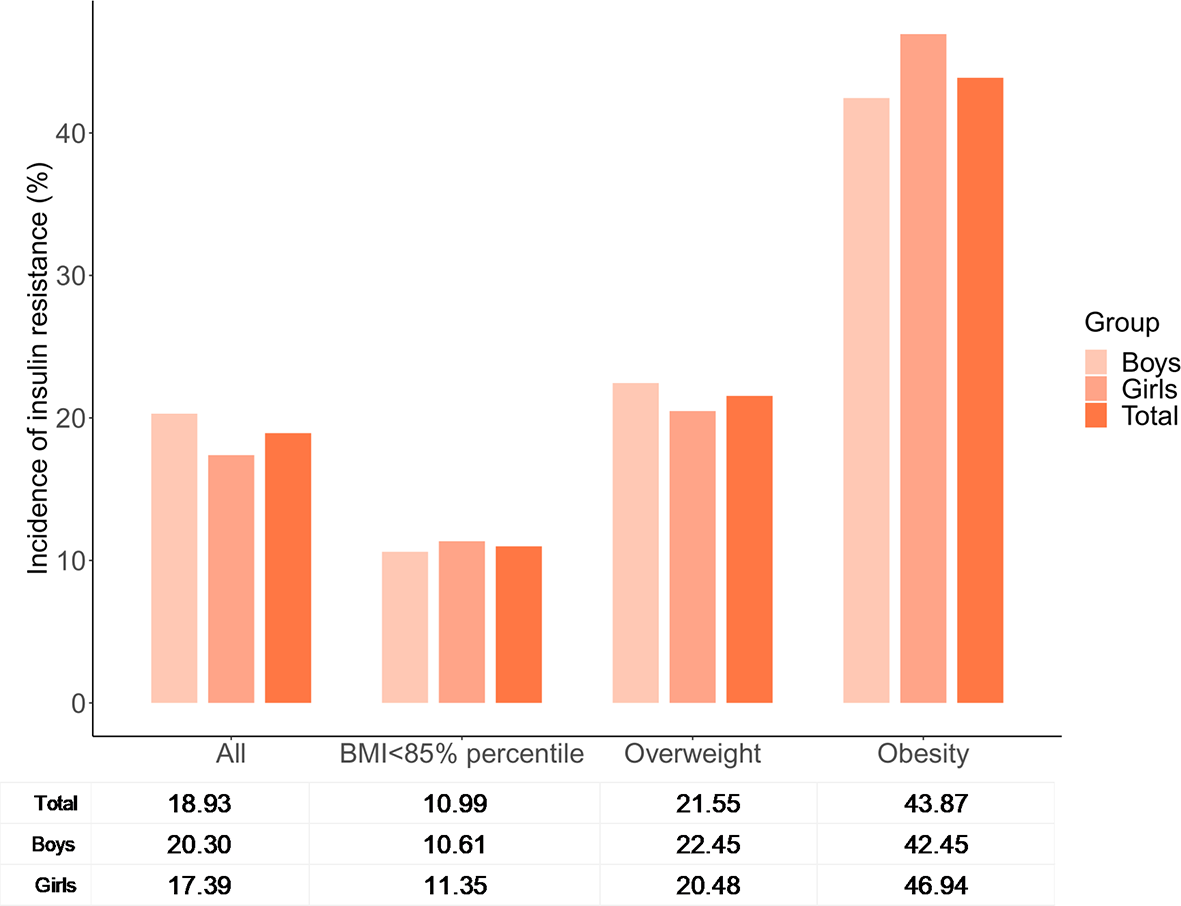
The incidence of insulin resistance based on body mass index categories.

A total of 167 adolescents were classified as having IR based on cutoff A (90th percentile of the HOMA-IR for adolescents with a normal BMI and FPG level within each sex group), with a prevalence of 18.93%. The prevalence of IR varied among different BMI categories (**Figure 2**), and was highest in adolescents with obesity (43.87%) and lowest in adolescents with a BMI of <85th percentile (10.99%). There were no significant differences in the prevalence of IR between boys and girls either in total or in each subgroup alone.

### Development of an Anthropometry-Based Predictive Nomogram for Insulin Resistance

**Figure 3:**
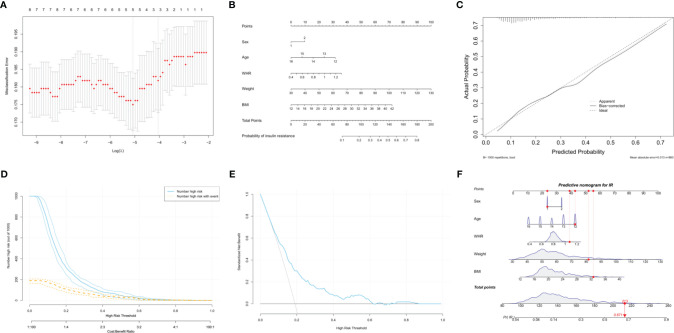
Development of an anthropometry-based nomogram for predicting insulin resistance among adolescents in China. **(A)** The LASSO coefficient profiles of eight variables. The coefficient profile plot was produced against the log(lambda) sequence. Five features with nonzero coefficients were selected by optimal lambda (0.006259), including sex, age, waist-to-hip ratio (WHR), weight and body mass index (BMI). **(B)** An anthropometry-based nomogram for the prediction of insulin resistance (IR) among adolescents in China, that includes sex, age, WHR, weight and BMI. **(C)** The calibration analysis. The x- and y-axes represents the nomogram-predicted probability of IR and the actual rate of IR, respectively. The solid line represents the performance of the nomogram. A more favorable performance is indicated by a closer fit to the ideal calibration line (the diagonal dotted line). **(D)** The clinical impact curve for evaluating clinical use. **(E)** The decision curve for evaluating clinical use. **(F)** Dynamic nomogram. An adolescent was randomly selected from the population, and the risk of IR was predicted using the five anthropometric indices of the nomogram.

Overweight and obesity are often associated with IR. Weight, BMI, WC, WHR and WHtR are common anthropometric indices used to assess global or local obesity in individuals. Based on the LASSO regression analysis, WHR, weight, and BMI had nonzero coefficients in addition to sex and age, with the greatest predictive power for IR (**Figure 3A**). A model incorporating sex, age, WHR, weight, and BMI was developed based on logistic regression analyses and presented as a nomogram (**Figure 3B**). The probability of IR was accurately predicted using the calibration curve (**Figure 3C**). The clinical impact and decision curves (**Figures 3D, E**) revealed that our model demonstrated a positive net benefit without increasing the number of false positives.

A dynamic nomogram using a random participant was established to predict the risk of IR (**Figure 3F**). The participant’s characteristics were as follows: 12-year-old boy, WHR = 1.08, weight = 81.5 kg and BMI = 32.65 kg/m^2^. The total points were 213 and the probability of IR was 67.1%. Therefore, this tool provides an individualized estimate of the risk of IR and should be useful to patients and healthcare providers in the long-term management of adolescents with cardiometabolic risk. We also developed a dynamic nomogram using online software (https://doctordu.shinyapps.io/InsulinResistanceRiskNomogram/).

## Discussion

In China, the prevalence of overweight and obesity among children and adolescents has dramatically increased over the decades and in 2019, was 11.1% and 7.9%, respectively (29). With the earlier onset of obesity, it is also likely that the prevalence of IR has increased among children and adolescents, which may explain the concomitant earlier onset of pediatric metabolic disorders (2, 3, 30). Although IR has been extensively studied among adults (31, 32), there remains a lack of population-based studies on classifying IR in adolescents using specific criteria, prevalence, or the extent of influences on CMRFs.

Owing to the influence of pubertal development, it is imperative that the reference ranges used for diagnosis of IR in adolescents are appropriate for the relevant population. Currently, there are no universally agreed upon reference ranges that can be used to diagnose IR in adolescents. We summarized the main cut-off values of HOMA-IR for defining insulin resistance in children and adolescents in recent literatures (**Table 4**). Researchers in Turkey calculated the cut-off values for HOMA-IR by receiver operating characteristic (ROC) analysis, upon the IR criterion of a total blood insulin level exceeding 300 μU/mL during a standard oral glucose tolerance test (7, 38, 39). However, this method is not applicable for use in population-wide screening, owing to the relatively high cost and complexity. It is also inappropriate to apply their cutoff values directly in other races. Consequently, cutoff values for the diagnosis of IR have been defined based on the HOMA-IR distribution of a healthy reference population or the study population itself (19, 21, 33–37). The cutoff values of the HOMA-IR used for defining IR in adolescents varies widely according to ethnicity, age, gender, and screening methods, and ranges from 2.4 to 5.22 (19, 34, 36, 39). Due to the subjectivity and limitation in population studies of adding the Tanner criteria in identifying IR, there is a proposal to use cutoff values for HOMA-IR according to gender and pubertal staging (39). Here, we defined IR using the threshold of the 90th percentile of the HOMA-IR for adolescents with a normal BMI and FPG level within each sex group (5.64 for boys and 5.73 for girls), and the 75th percentile for adolescents of the same sex (5.24 for boys and 5.20 for girls), namely cutoffs A and B. A previous study evaluated the frequencies of dyslipidemia and hyperglycemia in children with IR assessed by cutoff points of HOMA-IR; the frequencies of increased TC, LDL-C and TG, decreased HDL-C were all above 50% (35). Another study demonstrated that in a sample of Korean children and adolescents, metabolic syndrome increased 18.4-fold and each MetS component (including central obesity, hypertriglyceridemia, hypertension, low HDL-C, and hyperglycemia) increased 2.12–7.42-fold among participants with IR (40). However, they used different cutoff values of HOMA-IR for the diagnosis of IR. Our study focused on more CMRFs and the clustering of CMRFs, and was not limited to only MetS components (36, 41). The partial correlation analysis revealed a significant association between the HOMA-IR and CMRFs, and HOMA-IR was more significantly correlated with WC, FPG, TG, and SUA compared with other CMRFs evaluated. The logistic regression, showed that IR was significantly associated with most CMRFs, except decreased HDL-C levels. Although, the direction of causality among IR and CMRFs could not be determined owing to the cross-sectional nature of this study, adolescents with IR have a 3.132- and 2.662-fold increased risk of clustering of CMRFs based on cutoffs A and B, respectively. Therefore, the early identification of IR is critical for early intervention and prevention of cardiometabolic disorders. The kappa coefficient (κ) suggested excellent agreement between cutoffs A and B for determining IR. However, based on cutoff B, IR was not correlated with hypercholesterolemia, and was associated with dyslipidemia only after adjustments for sex and age. Moreover, IR, as defined by cutoff A, was more closely associated with the clustering of CMRFs than IR as defined by cutoff B. Overall, the results suggested that cutoff A is the optimal indicator for clinical application. Using cutoff A, we further assessed the incidence of IR, which was 18.93% in this study, and increased from 10.99% to 43.87% based on the different BMI categories. This finding was similar to that of previous population-based studies (36, 37). Several studies have reported widely varying prevalence rates of IR among adolescents with obesity in different countries, such as the United States (52.1%) (33) and Brazil (29.1%) (42). Similar to other studies, there were no obvious differences based on sex in the frequency of IR (35, 40, 41).

**Table 4:** Main cut-off values of HOMA-IR for defining IR in children and adolescents in recent literatures.

Country	Sample size	Prevalence of IR	Population	Cut-off values	Criteria	Refs
America	1802	52.1% in obese adolescents	Adolescents without diabetes aged 12-19 years	4.39	2 SD above the average	(33)
Argentina	226		Healthy children and adolescents aged 1 to 18 years old	Prepubertal 1.9 (≤ 7.5years 1.4, > 7.5years 2.0), pubertal 2.5 (girls 2.6, boys 2.4)	97th percentile	(34)
Brazil	383	56.10%	Children and adolescents aged 7 to 17.9 years	Boys (7-8.9 years 1.76, 9-10.9 years 1.97, 11-12.9 years 2.65, 13-14.9 years 3.21, 15-17.9 years 2.39), girls (7-8.9 years 1.39, 9-10.9 years 2.62, 11-12.9 years 3.02, 13-14.9 years 3.46, 15-17.9 years 2.89)	2 SD above the average	(35)
Chile	1192		Children and adolescents with normal BMI and fasting blood fasting blood glucose aged 10-15 years	Tanner I and II (boys 3.2, girls 4.1), Tanner III and IV (boys 4.2, girls 5.0)	90th percentile	(19)
China	1037	44.3% in obese participants	Children and adolescents with normal weight status and without any components of metabolic syndrome aged 6 to 18 years	Total 3.0, prepubertal 2.6, pubertal 3.2 6-9 years 2.1, 10-18 years 3.2 Male 3.1, Female 2.9	95th percentile	(36)
China	831		Children and adolescents aged 7 to 18 years	Boys (prepubertal 2.94, pubertal 4.43, postpubertal 4.66), girls (prepubertal 2.62, pubertal 4.56, postpubertal 3.95)	75th percentile	(21)
Korean	2116	4.7%, 25.6%, and 47.1% in normal-weight, over-weight, and obese participants	Children and adolescents with normal BMI and fasting blood fasting blood glucose aged 10 to 20 years	Boys (10-11 years 3.7, 11-12 years 4.4, 12-13 years 4.52, 13-14 years 4.58, 14-15 years 5.01, 15-16 years 4.5, 16-17 years 4.08), girls (10-11 years 4.1, 11-12 years 4.87, 12-13 years 5.65, 13-14 years 5.45, 14-15 years 4.15, 15-16 years 4.09, 16-17 years 4.5)	95th percentile	(37)
Turkey	57	43.86%	Pubertal obese children and adolescents (mean age: 12.04 ± 2.90 years; mean BMI: 29.57 ± 5.53)	3.16	ROC curve	(7)
Turkey	148	37.16%	Obese children and adolescents (mean age: 10.86 ± 3.08 years, mean BMI: 27.7 ± 4.2)	2.7	ROC curve	(38)
Turkey	208	Prepubertal (boys 37%, girls 27.8%), pubertal (boys 61.7%, girls 66.7%)	Obese children and adolescents aged 5 to 18 years	Prepubertal (boys 2.67, girls 2.22), pubertal (boys 5.22, girls 3.82)	ROC curve	(39)

BMI, body mass index; IR, insulin resistance; ROC, receiver operating characteristic curve; SD, standard deviation.

Although several studies have been conducted to identify the anthropometric indices for IR among adolescents (42–44), few studies have integrated multiple anthropometric indices in a prediction model or scoring system to predict the individual risk of IR among adolescents. Nomograms, which allow for the seamless incorporation of risk prediction into clinical decision making, are widely used as personalized risk-prediction tools. According to the LASSO analysis, age, gender, BMI, weight, and WHR are important predictors of IR in adolescents. Our study quantified the above factors in a statistical prediction model, which we converted to a clinically usable nomogram that potentially illustrates the extent to which individual risk indicators contribute to the risk of IR. Its classifying power and clinical use were supported by the calibration, clinical impact and decision curves. As depicted in our example, the probability of a 12-year-old boy with a WHR of 1.08, weight of 81.5 kg, and BMI of 32.65 kg/m^2^, having IR is 67.1%. This was the first study to develop an anthropometry-based nomogram for predicting individualized probabilities of IR among adolescents in China.

This study has some limitations. The Tanner stage data, which may be necessary for accurately classifying adolescence, were not included. Owing to the limited sample size, we were unable to stratify the analyses by age and pubertal stage (early, mid, and late puberty). Further research focused on the association of IR with other CMRFs (such as nonalcoholic fatty liver disease) among adolescents are awaited to make the results more valuable for reference. Our study included a single-center cohort, which was not representative of the entire adolescent population in China. Further evaluation in large-scaled, multicenter-study populations is imperative to generalize these findings.

In conclusions, IR, as defined by the HOMA-IR, was significantly associated with an increased cardiometabolic risk. For defining IR among adolescents in China, the optimal thresholds, using the HOMA-IR, were above 5.64 for boys and 5.73 for girls. The incidence of IR increases with an increased BMI, and is highest among adolescents with obesity. Additionally, we developed an anthropometry-based nomogram for predicting IR among adolescents, that incorporated sex, age, WHR, weight and BMI, and which may facilitate health counselling, self-risk assessments and early detection of IR.

## Data Availability Statement

The processed data that support the findings of this study are available from the corresponding author upon reasonable request.

## Ethics Statement

The studies involving human participants were reviewed and approved by Ethics Committee of Liaoyang Diabetes Hospital. Written informed consent to participate in this study was provided by the participants’ legal guardian/next of kin.

## Author Contributions

RD and YW conceptualized the study, carried out the initial analyses, and drafted the initial manuscript. PL and LL designed the data collection instruments, collected data, and revised the manuscript. All authors have read and agreed to the published version of the manuscript.

## Funding

The study was supported by the scientific and technological planning project of Liaoning Province (Grant 2008225009-21).

## Conflict of Interest

The authors declare that the research was conducted in the absence of any commercial or financial relationships that could be construed as a potential conflict of interest.

## Publisher’s Note

All claims expressed in this article are solely those of the authors and do not necessarily represent those of their affiliated organizations, or those of the publisher, the editors and the reviewers. Any product that may be evaluated in this article, or claim that may be made by its manufacturer, is not guaranteed or endorsed by the publisher.
